# No fate but what we make: a case of full recovery after out-of-hospital cardiac arrest

**DOI:** 10.1186/1757-7241-17-63

**Published:** 2009-12-11

**Authors:** Mafalda Miranda, Pedro J Sousa, Jorge Ferreira, Maria J Andrade, Pedro A Gonçalves, Cristina Romão

**Affiliations:** 1 Anesthesiology Department, Hospital Curry Cabral, Lisbon, Portugal; 2 Cardiology Department, Hospital de Santa Cruz, Carnaxide, Portugal

## Abstract

An 80 years old man suffered a cardiac arrest shortly after arrival to his local health department. Basic Life Support was started promptly and nine minutes later, on evaluation by an Advanced Life Support team, the victim was defibrillated with a 200J shock. When orotracheal intubation was attempted, masseter muscle contraction was noticed: on revaluation, the victim had pulse and spontaneous breathing.

Thirty minutes later, the patient had been transferred to an emergency department. As he complained of chest pain, the ECG showed a ST segment depression in leads V4 to V6 and laboratorial tests showed cardiac troponine I slightly elevated. A coronary angiography was performed urgently: significant left main plus three vessel coronary artery disease was disclosed.

Eighteen hours after the cardiac arrest, a quadruple coronary artery bypass grafting operation was undertaken. During surgery, a fresh thrombus was removed from the middle left anterior descendent artery. Post-operative course was uneventful and the patient was discharged seven days after the procedure. Twenty four months later, he remains asymptomatic.

In this case, the immediate call for the Advanced Life Support team, prompt basic life support and the successful defibrillation, altogether, contributed for the full recovery. Furthermore, the swiftness in the detection and treatment of the acute reversible cause (myocardial ischemia in this case) was crucial for long-term prognosis.

## Introduction

Cardiac arrest is an important cause of death and it is estimated that about 50 percent of those deaths occur outside hospitals [1].

The overall rate of successful resuscitation in patients with out-of-hospital cardiac arrest has been poor [1-3], with time to defibrillation being the most important factor for the success [2,4-8]. Basic life support improves survival by delaying the degradation of the cardiac rhythm to asystole, enhancing the possibility of successful defibrillation [5].

We describe a case of a successful resuscitation after an episode of sudden cardiac arrest, in an old patient with undiagnosed severe coronary artery disease and presumable acute coronary syndrome. Written informed consent was obtained from the patient for publication of this case report and any accompanying images.

## Clinical report

An 80 years old man, with history of hypertension and benign prostatic hypertrophy noted chest pressure for mild efforts and, some weeks later, he addressed to his General Practitioner for an appointment. After arriving to the Community Health Department he suffered a cardiac arrest. Immediate Basic Life Support (BLS) was started, with chest compressions and bag mask ventilation, and a request for an Advanced Life Support (ALS) team was made through the national emergency number (112).

**Figure 1:**
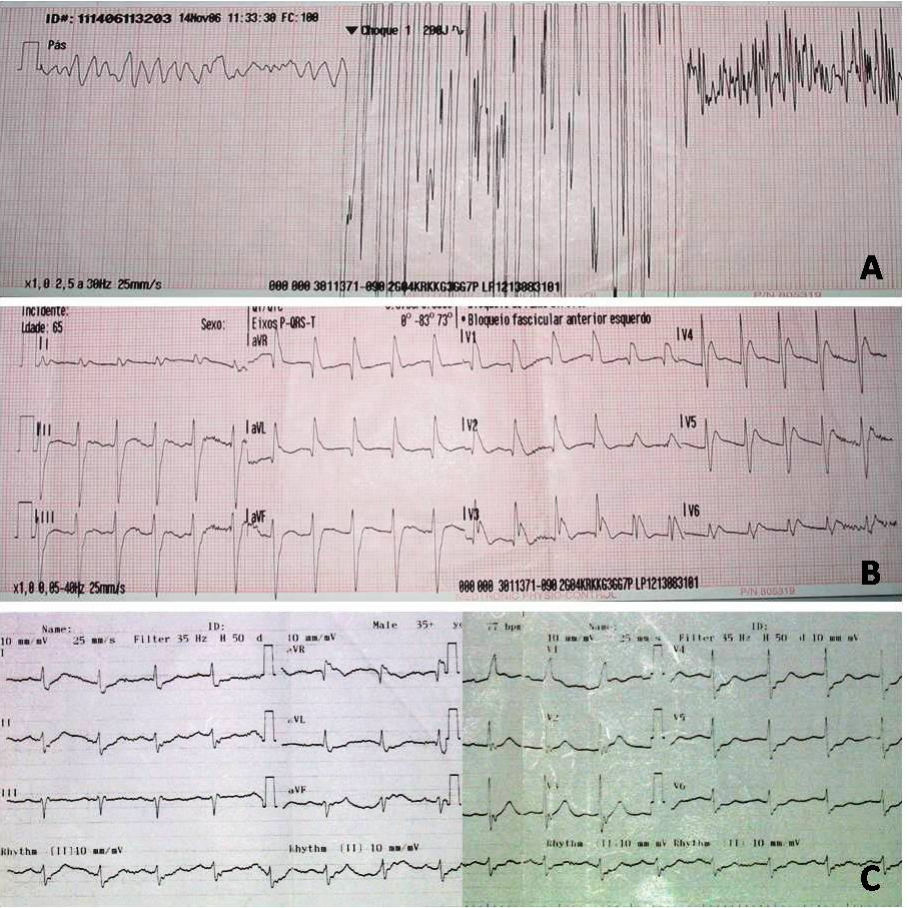
**Evolution of patient's cardiac monitorization and 12 lead ECGs**. A - Emergent cardiac monitoring revealing ventricular fibrillation and defibrillation with 200J. B - First 12 lead ECG after return of spontaneous circulation revealing a wide QRS tachycardia. C - Twelve lead ECG in the emergency department in sinus rhythm, heart rate of 75 bmp, right bundle branch block and ST-segment depression in leads V4-V6.

The initial evaluation made by the ALS team, about nine minutes after contact, confirmed the cardiac arrest in ventricular fibrillation. Defibrillation with 200J was performed (Fig. 1A) and BLS was continued.

When an endotracheal tube was to be inserted, right after defibrillation, the patient presented masseter muscle contraction, so BLS procedures were discontinued for reevaluation. The rhythm was a wide QRS tachycardia with pulse (Fig. 1B) and the victim had regained spontaneous breathing. The examination revealed blood pressure of 133/62 mmHg, heart rate of 130 bpm and pulse oximetry of 97% with an inspiratory oxygen fraction of 50%. There was a partial regain of consciousness, with a Glasgow Coma Score of 11 (eyes opening: 4, verbal response: 2, motor response: 5).

Thirty minutes after the event, the patient had been transferred to an emergency department. He remained hemodynamically stable without vasoactive support, with blood pressure of 96/50 mmHg, heart rate of 83 bpm, and pulse oximetry of 97%. Cardiopulmonary auscultation was normal.

There was a rapid improvement of the neurologic status (Glasgow Coma Score 15, with lacunar amnesia for the event) but the patient complained of chest pain. The electrocardiogram showed sinus rhythm with heart rate of 75, right bundle branch block and ST segment depression in leads V4-V6 (Fig. 1C).

Blood tests showed: creatine kinase (CK) 280 U/L, MB fraction 99 U/L, myoglobin 736 μg/L, cardiac troponin I 0.78 μg/L and CK-MB mass 6.7 μg/L.

**Figure 2:**
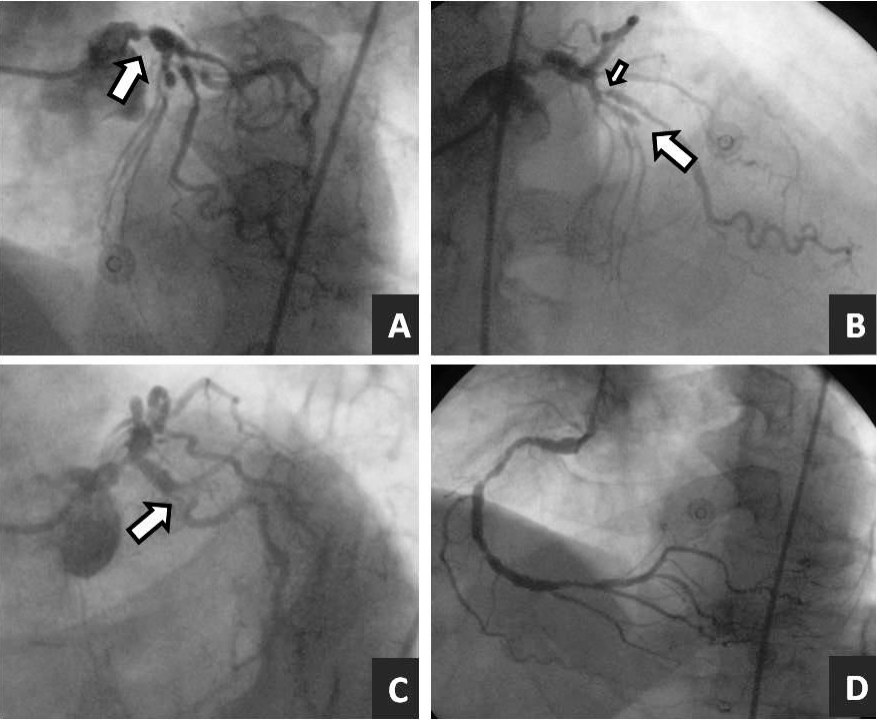
**Coronary angiography with a 50-70% stenosis in the left main coronary artery (arrow in A), occlusion in the middle left anterior descending artery (arrow in B), 90% ostial stenosis in the first diagonal (small arrow in B), 70-90% stenosis in the circumflex artery (arrow in C), and 70-90% stenosis in the middle and distal segments of right coronary artery (D)**.

An urgent coronary angiography revealed left main plus three vessels coronary artery disease with an occlusion in the middle left anterior descending (LAD) coronary artery. (Fig. 2).

The patient was referred to a cardiothoracic surgery center and was submitted to urgent coronary artery bypass grafting, which was performed 18 hours after the cardiac arrest. Myocardial revascularization was obtained with four aortic coronary bypasses with left internal mammary artery to LAD, an inverted saphenous vein segment to diagonal branch, and a sequential saphenous vein segment to second marginal and posterior descending arteries.

During the surgery a fresh thrombus was removed from the proximal segment of the LAD, which was probably the cause of the ventricular fibrillation. There were no complications after surgery, and the patient was discharged seven days later.

At 24-months follow-up the patient was asymptomatic and free from clinical events.

## Discussion

In this case, an elderly man with undiagnosed severe coronary artery disease was successfully resuscitated after cardiac arrest (CA). CA is defined as cessation of cardiac mechanical activity as determined by the absence of a palpable central pulse, apnea, and unresponsiveness [4]. Sudden CA is an important cause of death, being responsible for almost half a million deaths per year in the United States [1].

Although the majority of cases of sudden death from cardiac causes involve patients with preexisting coronary heart disease, CA is the first manifestation of this underlying problem in 50 percent of patients [2].

The overall rate of successful resuscitation in patients with out-of-hospital CA has been poor, averaging 2 to 23 percent in different reports [1-3].

About half patients with out-of-hospital CA are found in ventricular fibrillation or pulseless ventricular tachycardia [5]. In this subgroup of patients, when successfully resuscitated, between 21 and 34 percent were discharged alive from the hospital. One-year survival rates varied between 16 and 30 percent [2,3,6].

By far, the most important factor for success in resuscitation is time to treatment, in particular, defibrillation [2,4-8]. Obviously, this is also true for out-of-hospital CA, with better survival achieved with early defibrillation (less than four to eight minutes) [1,3,8], as the effectiveness of this procedure diminishes rapidly as time passes [9]. Each minute that defibrillation is delayed reduces by seven to ten percent the chance of hospital discharge. Resuscitation efforts initiated after eight to ten minutes are usually doomed to fail [2,5]. On the other hand, if CA from ventricular tachycardia or ventricular fibrillation occurs where there are readily available defibrillators (emergency room or automatic external defibrillators in out-of-hospital CA) the odds of survival is above 50% [10].

Other factors associated with better survival were early access to emergency medical care and early cardiopulmonary resuscitation [1,5-7]. BLS improves survival by delaying the degradation of the cardiac rhythm to asystole and in doing so enhances the possibility of successful defibrillation on arrival of the ALS team [5].

In the present report, the presence of the patient in a health care facility allowed the provision of BLS that raised the chance of recovery. Although the duration of CA has been estimated in about nine minutes, several features were identified as good outcome predictors. Considering the arrest itself, the recovery of effective spontaneous circulation after a short period of the ALS, without adrenaline (epinephrine), and the need of only one shock, indicated a good outcome [6,11]. The stability of hemodynamic status without inotropic or vasoactive support and the absence of hypotension, oliguria or hyperglycaemia were also indicative of good prognosis [11]. The early presence of a cough/swallow response (<30 minutes), pupillary light reflex (<2 minutes) and a Glasgow Coma Score of 10 or more (in the first 48 h) are also signs associated with better chances of recovery [11].

Following resuscitation from cardiorespiratory arrest, about 80 percent of patients remain comatose [11]. Although we found no reports, we believe that in out-of-hospital CA, if ALS is not readily available, the need for mechanical ventilation after return of spontaneous circulation is the rule. In the described case, the patient recovered breathing immediately after defibrillation. This was unexpected, considering that CA lasted for about ten minutes. Furthermore, the patient had reduced conscience (Glasgow Coma Score of 11), but he had a rapid recovery period and, about thirty minutes after the episode, he had Glasgow Coma Score of 15. There is evidence that induced hypothermia improves outcomes in patients who are comatose after resuscitation from out-of-hospital cardiac arrest [12,13]. With no persistent coma after return of spontaneous circulation, no therapeutic hypothermia was induced in this patient.

The early provision of BLS could have contributed to this rapid recovery. Also, at the initial evaluation the patient could be in ventricular tachycardia with low cardiac output (considered as CA by the first responder). This rhythm may have degenerated in ventricular fibrillation closer in time with the arrival of the ALS team. There are documented reports of similar evolutions [2].

The costs-effectiveness of out-of-hospital ALS is difficult to calculate. In a report describing the costs of out-of-hospital CAs of cardiac origin [14], the cost per patient discharged alive was €40 642, with a cost of €6632 per life year gained. Moreover, 4.4% of the costs were spent on patients not surviving to hospital, 35.6% on patients dying in the hospital while 60% of the total costs were spent on patients discharged from hospital alive [14].

## Conclusion

This case illustrates the possibility of long-term survival without neurological sequelae after an episode of sudden CA, in an old patient with undiagnosed severe coronary artery disease and presumable acute coronary syndrome. Although ALS was started nine minutes after the witnessed collapse, return of spontaneous circulation after the first defibrillation and prompt breathing recovery were positive predictors of the success of the resuscitation maneuvers. The fact that CA has occurred in a health care facility allowed prompt BLS, which contributed to the recovery. Furthermore, early detection and treatment of the acute reversible cause (myocardial ischemia in this case) was crucial for long-term prognosis.

## Consent

Written informed consent was obtained from the patient for publication of this case report and any accompanying images. A copy of the written consent is available for review by the Editor-in-Chief of this journal.

## Competing interests

The authors declare that they have no competing interests.

## Authors' contributions

MM was the case manager. MM and PJS conceived the case report and its design. PAG, MJA and CR helped draft the manuscript and added significant revisions. JF has revised and corrected the manuscript and given final approval for the publication. All authors read and approved the final manuscript.
